# Development and Assessment of a Body Condition Score Scheme for European Bison (*Bison bonasus*)

**DOI:** 10.3390/ani8100163

**Published:** 2018-09-26

**Authors:** Luisa Zielke, Nicole Wrage-Mönnig, Jürgen Müller

**Affiliations:** 1 Leibniz Institute of Zoo and Wildlife Research, Alfred-Kowalke-Straße 17, 10315 Berlin, Germany; 2 Grassland and Forage Sciences, Faculty of Agriculture and the Environment, University of Rostock, Justus-von-Liebig-Weg 6, 18059 Rostock, Germany; nicole.wrage-moennig@uni-rostock.de (N.W.-M.); juergen.mueller3@uni-rostock.de (J.M.)

**Keywords:** *Bison bonasus*, body condition score, score scheme, health status, rewilding, management

## Abstract

**Simple Summary:**

Europe’s largest terrestrial mammal, the European bison (*Bison bonasus*), has been successfully restored after the species had become extinct in the wild. In various reintroduction projects, captive bred European bison have been released into different habitats. Vigorous monitoring efforts are necessary to document how well the animals adapt to their new environment. In this study, we present a scheme that was developed for the observation of the body condition of adult European bison. Unbiased people with different professional backgrounds were asked to apply this scheme. While additional research is necessary to further validate the scheme, it was easy to use and covered the essential body traits. Therefore, it can be a helpful management tool.

**Abstract:**

Resettlement projects of the strongly threatened European bison (*Bison bonasus*) require a monitoring phase to assess both population status and habitat quality. Schemes of animal body condition scores (BCS) are robust tools to meet this requirement in practice. However, so far, no BCS scheme has been designed for European bison. Here, we suggest a body condition score scheme based on the extent of soft tissue around bony structures. The scoring system was developed with scores ranging from 1 (emaciated) to 5 (obese). Condition scores can be deduced after visually assessing the European bison both from the side and behind. Robustness of the scheme was evaluated: Unbiased people from different professional backgrounds were asked to assess the BCS of photographed semiwild European bison under field conditions and results were compared. Results demonstrate the suitability of the method. Nevertheless, variability of the results among assessors illustrates the necessity for training as well as for further research to validate the scheme as a true measure of physiological condition. We discuss the prospects and limits of a broad use of this scheme within the European bison community, and recommend the BCS scheme as a management tool.

## 1. Introduction

Currently, multiple projects are aimed at the resettlement of threatened European bison (*Bison bonasus*) in European countries like Poland [1], Denmark [2], Romania [3], and Germany [4], and the number is growing. These activities regularly include a transfer of bison reared in captivity into the wild. The associated change of diet from an offered ration to natural browsing and grazing pose a major challenge for the animals. Thus, monitoring of the nutritional status of the bison is necessary, especially in the case of newly introduced individuals or in poor habitats.

Body condition scores (BCS) are widespread measures for monitoring animal status in practice in wildlife management [5,6]. They are used either as surrogates for fitness-related traits or as a proxy for habitat quality, and are of key interest in studies of herbivore behaviour [7,8,9,10,11] and in ecology and evolution [12]. The relationship between subjective BCS and quantitative measures like thickness of rump fat, subcutaneous lipid content, and other physiological trains has been examined in large mammalian herbivores, like white-tailed deer (*Odocoileus virginianus*) [11,13], elk (*Cervus elaphus*) [11,14,15], moose (*Alces alces*) [11], caribou (*Rangifer tarandus*) [16], and Asian elephants (*Elephas maximus*) [17]. However, few studies focus on the genus *Bison*. Ranglack and du Toit [18] investigated the performance of American bison (*Bison bison*), a close relative of European bison, in relation to habitat quality. They followed a visual condition scoring scale originally developed for African buffalo (*Syncerus caffer*) by Prins [19]. Vervaecke et al. [9] used a BCS scheme that was developed for caribou (*Rangifer tarandus*). The Government of Alberta [20] presented a body condition scheme for American bison (*Bison bison*) adapted from a beef and dairy cattle five-point scale. In the bison rewilding plan [21], a simple scheme based on visual clues of European bison is mentioned, but neither validated nor published. The absence of a species-specific BCS table for *Bison bonasus* is an obstacle to research and hampers data exchange among the different institutions involved in European bison resettlement and protection. Therefore, the aim of this work was to develop a BCS scheme that would both be easy to handle and reliable. In the following, we introduce a non-invasive, visually based five-point scale to evaluate the body condition of European bison in the wild and in captivity.

## 2. Materials and Methods

### 2.1. Principles of Scheme Design

Even though European bison are not domesticated, their genetic background [22] and morphology [23] is similar to domestic cattle *Bos taurus*, and the livestock sector has considerable experience with BCS schemes [24,25]. Today, the entire fertility and welfare management in professional dairy herds is based on such condition estimates. Thus, a BCS scheme for domestic cattle seemed a good starting point for developing a BCS scheme for European bison. A basic requirement of the scheme design was robustness in application, such that people not very familiar with external assessments are able to apply a scheme without expensive training [26]. This implies that the scheme should be based on few clearly detectable characteristics rather than on many difficult to assess features. Parts of the body that have to be evaluated should be easily visible by the assessor. European bison are difficult to handle and palpation is usually not an option, so the scheme should be based on visual assessment.

### 2.2. Scheme Design and Handling

**Figure 1:**
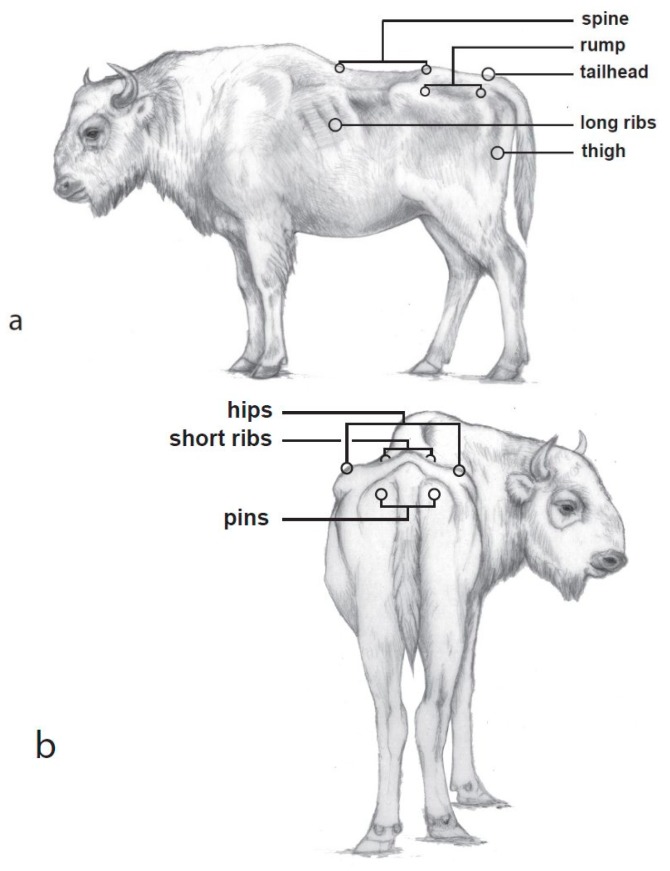
Key areas for assessing body condition score in European bison (*Bison bonasus*). (**a**) Left side view; (**b**) back view. Area names are consistent with the corresponding body condition score (BCS) labels.

In order to develop the BCS system, animals of both sexes, ranging from emaciated to obese, under semifree and zoo conditions, and older than 4 years were observed and photographed to analyse the possible range of body condition for the species. The resulting scheme is based on visible depressions around bone structures when viewed from the left side and from behind. For the BCS system, eight key body characteristics were evaluated for differences in soft tissue in order to determine which areas are most informative for field observation purposes. This information was then used for a simplification of the scheme. Viewed from the left, the spine, rump, tailhead, long ribs, and thigh are body regions easy to detect (Figure 1a). From behind, the hips, short ribs, and pins comprise the regions for assessing soft tissue (Figure 1b).

The resulting 5-point scale ranges from 1 (emaciated) to 5 (obese) (Figure S1). In the application of the BCS scheme, each key body area is scored separately. The average of the area scores per animal is the body condition score of this animal. Animals with a rounded appearance will be scored with a 5, because fat fills all possible regions and no bone structures are visible. With decreasing fat deposition, bone structures become more pronounced. Thin animals with a body condition score of 1 and 2 are characterized by deep depressions alongside bone structures. Animals with an even distribution of body fat should receive a score of 3.

### 2.3. Scheme Evaluation

Ten adult European bison were photographed from the left side and from behind (Figure S2). The aim was to take pictures from animals with different body condition statuses, but as the studied animals were living semifree, visibility of the animals was also an issue. Based on the pictures, the ten animals were scored by unbiased people with different professional backgrounds. Assessors were grouped into the following categories: (I) students with an interest in animal science, but with limited experience in body evaluation; (II) veterinarians; (III) animal experts with a lot of experience in various animals but not familiar with bison; (IV) cattle experts with a lot of experience with BCS systems but not familiar with bison; (V) key European bison experts. Each category was represented by five assessors. The assessors scored the photographs independently and without assistance. Results were evaluated concerning agreement between (categories of) assessors and the accordance of the single characteristics’ scores for the different individuals. Participants of the evaluation were additionally asked to comment on advantages and disadvantages of the scoring system based on their experiences in the scoring process. Based on the strong correlation between some of the key areas as well as the evaluation of the applicability of each trait by the assessors, the scheme was in the following simplified without a decisive loss of information.

### 2.4. Statistical Analyses

We used a mixed model fitted by restricted maximum likelihood approach (REML) to analyse the effects of assessors’ category and score regions at the animal level on the BCS. Assessors’ categories were regarded as fixed effects, whereas the body score regions were nested in the individual scored animals and treated as random effects. Satterthwaite approximation allowed us to perform an *F*-Test to check the estimates of the fixed effects for significance. Random effects were tested by the Chi square test. Non-parametric density estimation was used to illustrate the frequencies of the overall scores per animal assigned by assessors of different categories. Differences among assessors’ category score means were tested by Tukey contrasts with Bonferroni–Holm correction of all pairwise multiple comparisons at *p* = 0.05. Assessors’ agreements in scoring different body regions were analysed by intraclass correlation coefficients (ICC), according to Shrout and Fleiss [27], by means of bivariate Spearman’s rho rank correlations, and Kendall’s coefficient of concordance Wt [28]. We used the R package “irr” [29] for all concordance statistics.

Kendall’s tau was used to analyse the correlations between the single score regions, as suggested by Hollander and Wolfe [30]. To visualize the relation patterns of the body region scores, including the overall scores per animal, a multidimensional scaling was conducted and a cluster–network plot created, according to Jackson [31]. All statistical computing procedures were written in scripts of the statistical environment R [32].

## 3. Results

### 3.1. General Results of the Scheme Application

There was a significant effect of both the assessors’ category (*F*-Test, *F*-value 16.01, *p* < 0.001) and the influence of body score regions, nested in bison individuals (Chi square 1050, *p* < 0.001), on the BCS (see below). In all categories, some of the assessors mentioned that a light condition as well as movement of the animal can have an influence on the visibility of body characteristics and therefore on the scoring result. Additionally, it was noted that the evaluation of animals with a thick winter coat could be challenging. Some of the assessors commented on difficulties in evaluating the pins, short ribs, and tailhead.

### 3.2. Importance of the Assessors’ Professional Background

**Figure 2:**
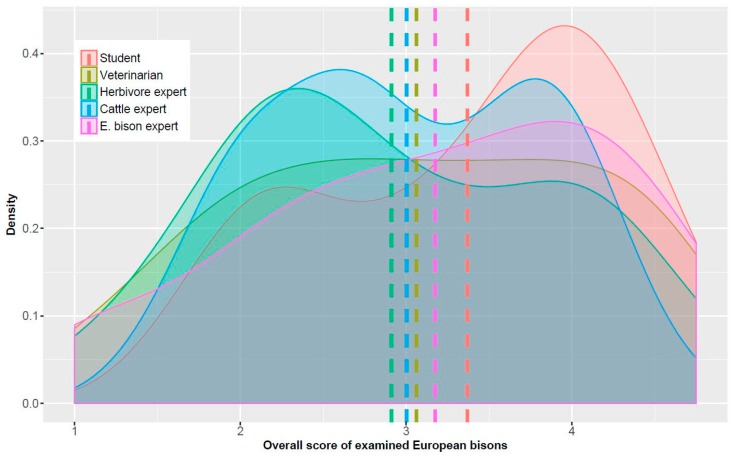
Density plot presenting frequencies of the overall scores per animal assigned by the assessors of different categories with different professional backgrounds. Dashed lines show the mean of the overall scores per category.

The distribution of given BCS of the five assessor categories as well as the mean score of each category is shown in Figure 2. Cattle experts obviously tended to orient themselves on the intermediate shape of the body condition to adjust their score level. It resulted in a quasi perfect symmetric mean score of 3. The mean score of the results given by herbivore experts was slightly lower, whereas those of the categories veterinarian, European bison experts, and students tended towards higher-ranking scores (Figure 2). Assessors in the category students gave more high-ranking scores compared to the assessors of the other categories.

**Figure 3:**
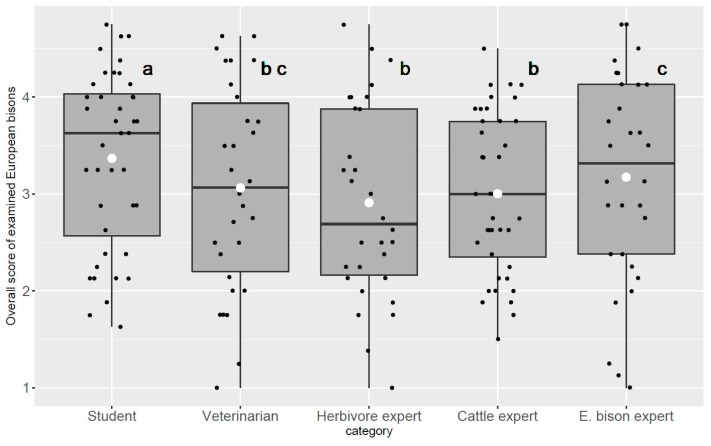
Box and whisker plots of the overall scores per animal per assessors’ category. The box is bounded on the top by the third quartile and on the bottom by the first quartile; the median divides the box; the whiskers represent observations outside the 9–91 percentile range. Raw data are dot-plotted as jitters. White dots indicate the means. Different letters in black indicate significant differences among category score means (Tukey Contrasts, *p* < 0.05; Bonferroni–Holm correction of all-pair multiple comparison).

In addition to the cattle experts, veterinarians also showed a tendency towards a homogenous distribution of their scores within the animal test group. This can be seen by the congruence between the mean and the median of the overall scores in Figure 3. Whereas cattle experts avoided excessive use of extreme maximum and minimum scores, the veterinarians and European bison experts tended to use the whole range of the rating scale (Figure 3). Irrespective of these tendencies, no significant differences in the mean BCS between the categories of veterinarians, herbivore, and cattle experts occurred. The largest agreement between assessors existed when evaluating animals with an even distribution of body fat (BCS near 3). Students showed the largest deviations from the other assessors’ categories when assessing bison with a poor body condition (Figure S3).

### 3.3. Role of the Score Regions

Correlation coefficients between the scores from different body regions ranged from 0.54 to 0.79, indicating a moderate relationship in general. Irrespective of assessors’ backgrounds, the backbone scores and the scores for short ribs showed the highest degree of correlation. In contrast, the relationship between tailhead scores and the scores for pins was not very pronounced. With the exception of the pins, all other correlation coefficients of single traits with the overall score varied only slightly from 0.74 to 0.79. The relationship between the single score traits and the overall score was confounded, as the overall score was derived as a mean of the single trait scores on the animal x assessor-level. Therefore, the results presented in the last column must be considered separately (Figure S4).

**Figure 4:**
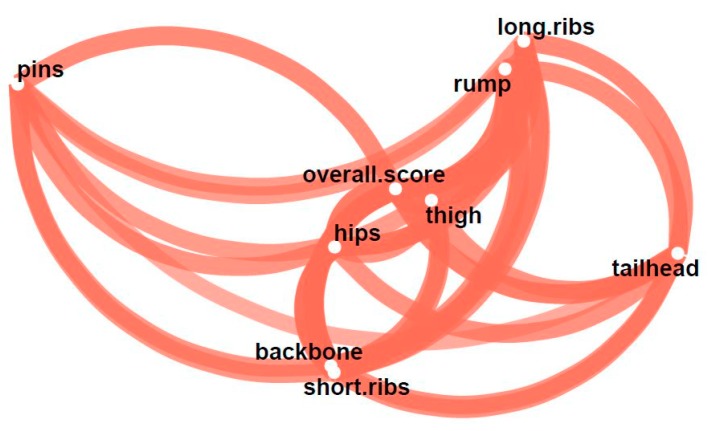
Score region cluster–network plot. The proximity of the score regions was determined using multidimensional clustering. The closer each characteristic is to each other, the higher the relationship, regardless of assessors’ backgrounds. Line thickness shows the correlation with the mean score value. All correlations were positive.

The score region cluster–network plot (Figure 4) uses clustering techniques to facilitate detection of closely related variables. The closer variables are arranged in space, the smaller their risk to differ regardless of assessors’ categories. For example, assessments of backbone and short ribs as well as those of long ribs and rump resulted in very similar scores, irrespective of assessors’ professional backgrounds and individual animal differences. By contrast, the widely spaced variables pins and tailhead were positioned as outsiders. This indicates that these regions are more sensitive to changes in assessors’ backgrounds and/or animals’ trait specification. In addition to the spatial nearness of the regions, the plot also shows the proximity to the mean score. The correlation illustrates another statistical test variable in the plot that is to be taken into account when selecting redundant regions. It is illustrated by the line width in the diagram.

The different statistical parameters to quantify assessors’ agreements in the overall score (see last row of Table 1) were used to derive information on the functionality of the scheme as a whole. All statistical approaches showed a high to very high level of agreement.

Turning to the single score traits (Table 1), some differences became obvious. Thus, the outlying variables (Figure 4) for pins and tailhead differed in their scores: The tailhead score showed fairly good consistency among assessors’ scores, while the pins score showed the weakest agreement (Table 1).

## 4. Discussion

**Figure 5:**
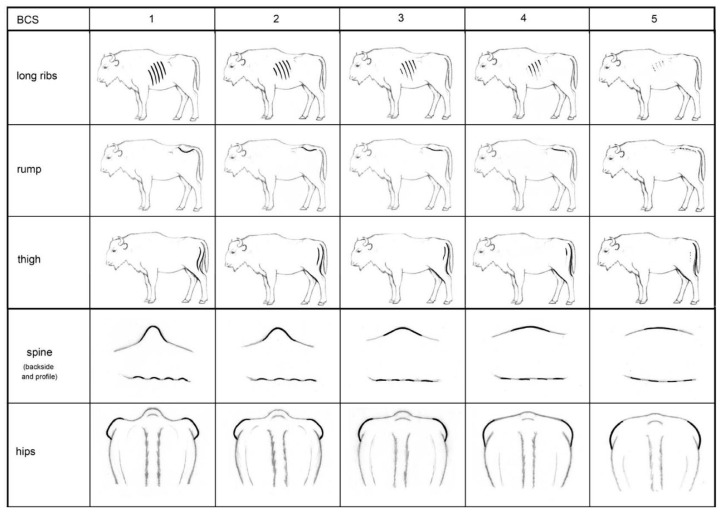
Simplified body condition score scheme for European bison (*Bison bonasus*) with five key areas and a five-point scale from 1 (emaciated) to 5 (obese).

This study presents the development of a BCS scheme for European bison. Further, the study tested the agreement among and within categories of scorers, showing that the scoring system itself is a robust measure. The results indicated that most of the key areas assessed were strongly correlated, which suggests that the scoring scheme could be simplified without losing much information. We therefore modified the scheme by reducing the key body areas to five. The highest degree of correlation within all assessors’ categories was between the spine and short ribs, suggesting that neighbouring regions may influence each other more than those located on different parts of the body. Due to the strong correlation between spine and short ribs, we decided to dispense with short ribs. Despite their proximity on the body, tailhead and pins showed the lowest degree of correlation between the scores within all assessors’ categories. To find a compromise between detail and observation time, we deleted both these areas, as some assessors also mentioned difficulties with these during the evaluation. The simplified scheme therefore consists of five areas: Long ribs, rump, thigh, spine, and hips (Figure 5).

The evaluation demonstrated that each of these was suitable for the evaluation of the body condition on its own. Thus, the combination of all features allows for a reliable assessment of the body condition. Assessing five key body areas is a compromise between a result as detailed as possible and the often limited time for observation and valuation of an animal in the wild.

Assessors who were unfamiliar with European bison did not differ strongly from experts on the species. We assume for the discussion that scores by bison experts were—due to their expertise with the animals—more accurate than those of other assessors’ groups, but this has so far not been validated. Cattle experts are familiar with BCS because it is a widely used tool for dairy cattle management [33,34]. Similarly, veterinarians are used to evaluating the health status and body condition of animals. Cattle experts and veterinarians demonstrated that familiarity with the use of BCS to evaluate body condition can compensate for a lack of bison-specific knowledge. Herbivore experts tended to give lower-ranking BCS compared to all other categories. Their results were possibly influenced by their experience with herbivores with different anatomical and physiological conditions, like horses or sheep. Even an obese bison will not attain the rounded rump that is common for horses [35]. Students were neither familiar with the application of BCS nor with European bison and showed the strongest deviations compared to the other categories. In order to obtain more consistent results, special training programmes for the assessors could be promising [8,36].

## 5. Conclusions

This study has presented a practical scheme to evaluate the BCS of European bison. The scheme can be applied while directly observing animals as well as by using photographs. We offer this BCS scheme to the European bison community and recommend further evaluation of the scheme, including correlations of BCS with body lipid content, subcutaneous fat, animal weights, shoulder heights and any other fitness related traits. The more information that is available about the assessed animal, the better the BCS can be interpreted [6,9,37].

## Figures and Tables

**Table 1:** Assessors’ agreement in scoring different body regions. Intraclass correlation coefficients (ICC), mean of bivariate Spearman’s rho rank correlations (Rho), and Kendall’s coefficient of concordance (Wt) were used as indices of inter assessors’ concordance.

Body Region (Score Traits)	ICC Type ‘Agreement’ (Unadjusted, *F*-Test)	ICC Type ‘Consistency’ (Adjusted, *F*-Test)	Spearman’s Rho (z-Test)	Kendall’s Wt (Chi Square Test)
long ribs	0.97 *** F(9, 45.8) = 70.7; *p <* 0.001	0.99 *** F(9, 144) = 70.7; *p <* 0.001	0.88 * z = 2.33; *p <* 0.05	0.87 *** Chisquare(9) = 133; *p <* 0.001
rump	0.97 *** F(9, 79.2) = 49.5; *p <* 0.001	0.98 *** F(9, 144) = 49.5; *p <* 0.001	0.81 * z = 2.14; *p <* 0.05	0.79 *** Chisquare(9) = 121; *p <* 0.001
tailhead	0.95 *** F(9, 71.8) = 33.1; *p <* 0.001	0.97 *** F(9, 144) = 33.1; *p <* 0.001	0.74 *^n.s.^* z = 1.95; *p =* 0.051	0.71 *** Chisquare(9) = 109; *p <* 0.001
thigh	0.96 *** F(9, 108) = 29.5; *p <* 0.001	0.97 *** F(9, 144) = 29.5; *p <* 0.001	0.71 *^n.s.^* z = 1.88; *p =* 0.060	0.68 *** Chisquare(9) = 104; *p <* 0.001
backbone	0.95 *** F(9, 91.9) = 28.7; *p <* 0.001	0.96 *** F(9, 144) = 28.7; *p <* 0.001	0.70 *^n.s.^* z = 1.85; *p =* 0.065	0.67 *** Chisquare(9) = 103; *p <* 0.001
short ribs	0.94 ***F(8, 88) = 21.4; *p <* 0.001	0.95 ***F(8, 128) = 21.4; *p <* 0.001	0.58 *^n.s.^* z = 1.41; *p =* 0.159	0.56 *** Chisquare(8) = 75.6; *p <* 0.001
hips	0.90 *** F(8, 76.3) = 14.4; *p <* 0.001	0.93 *** F(8, 136) = 14.4; *p<* 0.001	0.66 *^n.s.^* z = 1.62; *p =* 0.104	0.62 *** Chisquare(8) = 89.8; *p <* 0.001
pins	0.81 *** F(9, 62.2) = 8.34; *p <* 0.001	0.88 *** F(9, 144) = 8.34; *p<* 0.001	0.62 *^n.s.^* z = 1.63; *p =* 0.102	0.58 *** Chisquare(9) = 89.4; *p <* 0.001
all score	0.92 *** F(9, 69.7) = 18.6; *p <* 0.001	0.95 *** F(9, 144) = 18.6; *p <* 0.001	0.83 * z = 2.20; *p <* 0.05	0. 78 *** Chisquare(9) = 119; *p <* 0.001

*^n.s.^*: not specified; *: *p <* 0.05; ***: *p <* 0.001.

## Supplementary Materials

The following are available online at http://www.mdpi.com/2076-2615/8/10/163/s1, Figure S1: Body condition scheme (BCS) for European bison (*Bison bonasus*) with a five-point scale ranging from score 1 (emaciated) to score 5 (obese), Figure S2: Pictures of ten adult European bison (*Bison bonasus*) photographed from the left side and from behind, Figure S3: Box plots of the given scores per animal per assessors’ category; Figure S4: Kendall’s tau correlation matrix of all single trait scores including the derived overall score.
