# Supplementary material for: Development and Assessment of a Body Condition Score Scheme for European Bison (Bison bonasus)
Source: Animals (Basel). 2018 Sep 26;8(10):163. doi: 10.3390/ani8100163 (PMC6210254; doi:10.3390/ani8100163)
Supplement: Supplementary file 1 [file animals-08-00163-s001.pdf]

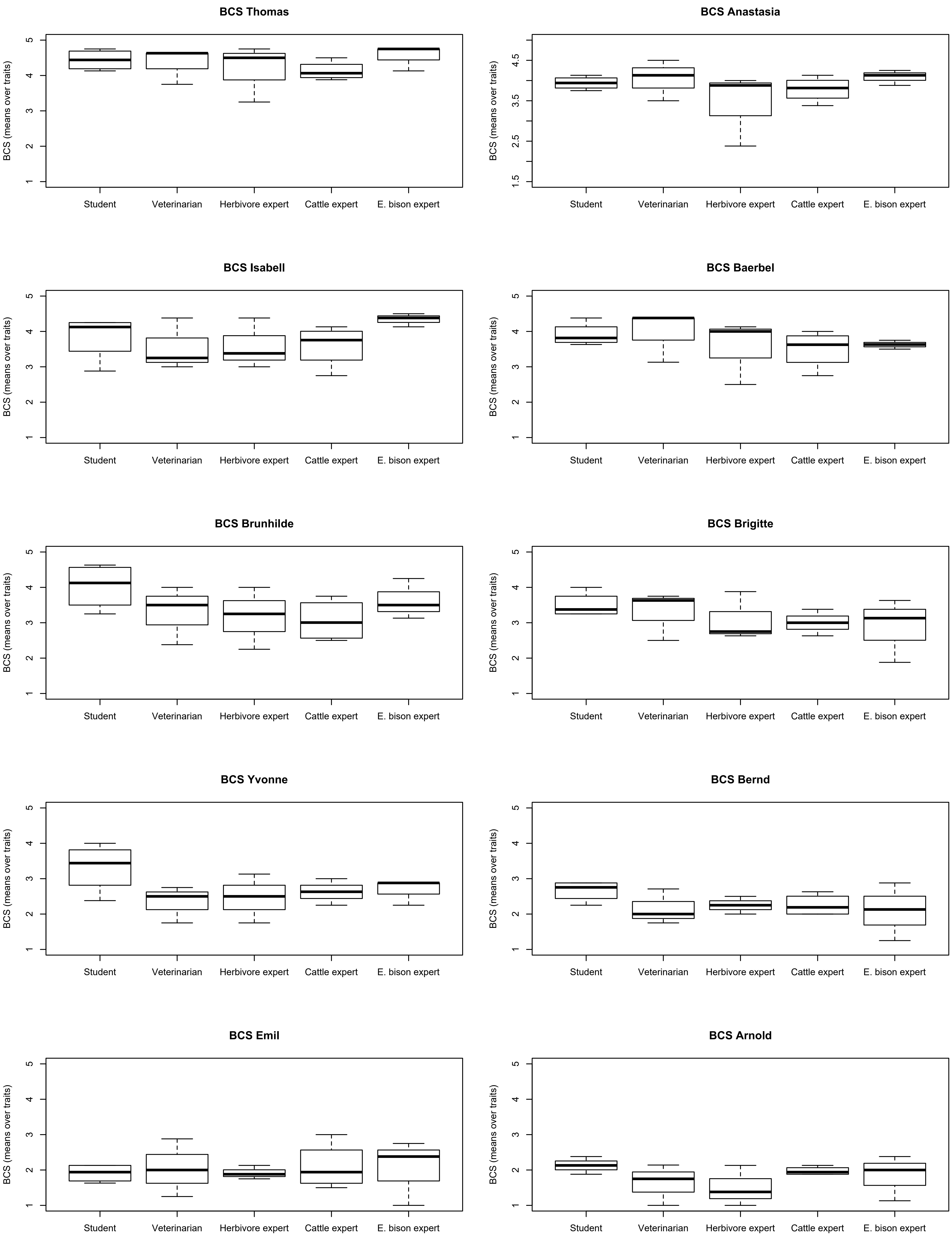

BCS Thomas
BCS Anastasia
BCS Isabell
BCS Baerbel
BCS Brunhilde
BCS Brigitte
BCS Yvonne
BCS Bernd
BCS Emil
BCS Arnold
BCS (means over traits)
1
2
3
4
5
1.5
2.5
3.5
4.5
Student
Veterinarian
Herbivore expert
Cattle expert
E. bison expert
